# Lattice strain relaxation in thin Mo films grown heteroepitaxially on MgO single crystals

**DOI:** 10.1107/S1600576726000233

**Published:** 2026-02-01

**Authors:** Petr Cejpek, Mykhaylo Motylenko, David Rafaja

**Affiliations:** a Institute of Materials Science, TU Bergakademie Freiberg, Gustav-Zeuner-Str. 5, D-09599Freiberg, Germany; Institut de Recherche sur les Céramiques, France

**Keywords:** heteroepitaxy, thin films, orientation relationships, lattice misfit, stress relaxation, X-ray diffraction, crystallite group method, transmission electron microscopy, electron diffraction

## Abstract

Mechanisms of the lattice strain relaxation in molybdenum thin films that were grown heteroepitaxially on (001)- and (011)-oriented MgO wafers using magnetron sputtering were studied using a combination of X-ray and electron diffraction and transmission electron microscopy. For the Mo film grown on (001)-oriented MgO, the X-ray diffraction pole figure measurements revealed (001)_Mo_ ∥ (001)_MgO_ & [110]_Mo_ ∥ [100]_MgO_ as the main orientation relationship. On the (011)-oriented MgO, the Mo film grew with the orientations (112)_Mo_ ∥ (011)_MgO_ & ±[110]_Mo_ ∥ [100]_MgO_. In all cases, the stress generated by the lattice misfit exceeded the elastic deformation limit of Mo, which activated the lattice strain relaxation mechanisms, mainly the formation of dislocations and slip and twinning on the lattice planes {112}. The dominant relaxation mechanism depends on the mutual orientation between the film and the substrate, which defines the direction of the deformation force in the film. In the (001)-oriented film, the lattice strain produced by the lattice misfit was reduced by twinning and dislocations. In the film having the (112) orientation, the main relaxation mechanism was the formation of dislocations. In both cases, the deformation energy was additionally reduced by the small lateral size of the Mo crystallites.

## Abstract 2

The possibility of controlling the crystallographic orientation of thin metallic films via heteroepitaxy to a single-crystalline substrate is illustrated on the example of molybdenum films deposited on MgO substrates having the orientations (001) and (011). The relaxation mechanisms of the lattice strain induced by the lattice misfit between the film and the substrate were identified via X-ray and electron diffraction and electron microscopy.

## 1. Introduction

Heteroepitaxial growth of thin films on single-crystalline substrates is a very efficient tool for production of materials with specific crystallographic orientations (Grünbaum, 1975 ▸). The orientation of the films is typically controlled by the crystallographic symmetry operations of the substrates and by the minimum total deformation energy of the films (Geiesche *et al.*, 1988 ▸; Brune & Kern, 1997 ▸). As the heteroepitaxial films usually possess slightly different lattice parameters or even a different crystal structure from the substrate, the lattice misfit produces lattice strain that can be used for a targeted manipulation of materials properties via epitaxial strain engineering. Prominent examples of materials utilizing epitaxial strain engineering are multiferroics (Ramesh & Spaldin, 2007 ▸; Martin *et al.*, 2010 ▸; Dix *et al.*, 2010 ▸; Himcinschi *et al.*, 2010 ▸; Chernova *et al.*, 2015 ▸; Himcinschi *et al.*, 2015 ▸; Zhang *et al.*, 2018 ▸; Han *et al.*, 2020 ▸), AlGaN-based far-ultraviolet light emitting diodes (Knauer *et al.*, 2023 ▸) and high-electron-mobility transistors (Kang *et al.*, 2003 ▸; Rafaja *et al.*, 2021 ▸), thermoelectrics (Zhang *et al.*, 2023 ▸), and two-dimensional semiconductors (Chaves *et al.*, 2020 ▸). Heteroepitaxial growth of metallic thin films on non-metallic substrates is often employed to improve the mechanical properties of the metal–oxide interfaces (Ernst, 1995 ▸), in particular to enhance the adhesion of the films to the substrate (Rafaja *et al.*, 2009 ▸; Wüstefeld *et al.*, 2017 ▸; Drehmann *et al.*, 2018 ▸).

Mo thin films are frequently used as contacts in electronics (Yen *et al.*, 2007 ▸; Rafaja *et al.*, 2013 ▸; Sundarapandian *et al.*, 2025 ▸; Rahmouni *et al.*, 2025 ▸) and as back contacts in photovoltaic devices (Jubault *et al.*, 2011 ▸; Dhar *et al.*, 2013 ▸;Pandharkar *et al.*, 2018 ▸; Rashid *et al.*, 2019 ▸). The main reasons for these applications are the high electrical conductivity and good mechanical properties of Mo. However, these properties are strongly affected by the microstructure of the film. The electrical conductivity is deteriorated by almost all crystal structure defects, because they act as scattering centres for electrons, reduce the electron mobility and decrease the electrical conductivity (Hummel, 2011 ▸). The effect of point defects (substitutional and interstitial impurities), dislocations and grain boundaries on the electrical conductivity of thin Mo films was described by Rafaja *et al.* (2013 ▸). Pandharkar *et al.* (2018 ▸) studied the influence of morphology and preferred orientation of grains. Rahmouni *et al.* (2025 ▸) analysed the influence of preferred orientation and impurities. Sundarapandian *et al.* (2025 ▸) investigated in detail the effect of the high-angle grain boundaries and Σ boundaries, and concluded that highly symmetrical Σ boundaries scatter electrons less than less symmetrical grain boundaries.

In this study, we analyse the crystal structure defects and microstructure features that developed in thin Mo films deposited heteroepitaxially on single-crystalline MgO substrates having the orientations (001) and (011). The orientation relationships between Mo and MgO were deduced from pole figures measured using X-ray diffraction (XRD) and validated by selected area electron diffraction in a transmission electron microscope (SAED/TEM). Information about the crystal structure defects and other microstructure features was obtained from XRD measurements that were performed using the crystallite group method (Kužel *et al.*, 1994 ▸), complemented by convergent beam electron diffraction (CBED) and imaging in a TEM. Residual stress analysis using XRD revealed a significant relaxation of the lattice strain in the films. For the respective orientation relationship between the Mo film and the MgO substrate, the relaxation mechanisms were identified, and these are discussed in terms of growth twins, dislocations and anisotropy of the crystallite size.

## 2. Experimental details

Thin Mo films having a thickness of 100 nm were physical vapour deposited via magnetron sputtering (MS) on single-crystalline MgO wafers that had a diameter of 2.5 cm, a thickness of 0.5 mm, and the crystallographic orientation (001) or (011). The Mo targets used for the deposition had a purity of 99.95 wt%. The main impurities were oxygen (50 p.p.m.w.) and tungsten (<90 p.p.m.w.). Prior to the deposition, the deposition chamber was evacuated to 4 × 10^−4^ Pa. The deposition was carried out in argon, at a working gas pressure of 0.5 Pa and at a bias voltage of −80 V. The films were characterized using X-ray and electron diffraction and transmission electron microscopy.

The XRD experiments, which included pole figure measurements and 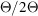 scans on selected crystallite groups, were carried out in a Bruker D8 Discover diffractometer that was equipped with a sealed X-ray tube with a Cu anode (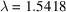
 Å) and with an Eulerian cradle. The primary beam was collimated by polycapillary optics. The diffracted intensities were measured by a scintillation detector. The angular acceptance of the detector (0.23°) was defined by a parallel plate collimator. The pole figures were measured for the Mo diffraction lines 110, 200 and 211 at sample inclinations (χ) between −75° and 75°. The step size of the sample inclination was 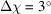. The step size of the sample rotation around its surface normal (φ) was 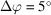.

The 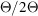 scans were measured for individual orientation variants using the crystallite group method (Kužel *et al.*, 1994 ▸). Respective angles χ and φ were selected on the basis of the pole figure measurements. In order to obtain positions and widths of individual diffraction lines, the XRD lines were fitted by pseudo-Voigt functions. These characteristics were utilized for analysis of the residual stresses, crystallite sizes and microstrain values. The instrumental line broadening (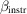) was corrected using a LaB_6_ standard (SRM 660c from NIST), which was measured at all available diffraction angles and at relevant χ inclinations. Individual dependences of 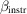 on 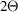 were fitted and interpolated using Caglioti polynomials (Caglioti *et al.*, 1958 ▸) for each sample inclination. As the XRD lines possessed a Gaussian shape, the instrumental line broadening was subtracted in the ‘quadratic’ form: 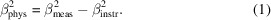


The XRD measurements were complemented by SAED, CBED and imaging in a TEM. All TEM experiments were carried out in a JEM 2200FS (JEOL Ltd, Japan), which was equipped with a field emission gun operating at 200 kV, a CESCOR probe aberration corrector (CEOS GmbH, Germany), an ultra-high-resolution objective lens (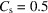
 mm), an in-column energy filter (Ω-filter), and a OneView camera (Gatan/AMETEK Inc., USA). The Ω-filter was used to filter inelastically scattered electrons and thus to improve the quality of the TEM images. The TEM samples were prepared by the focused ion beam (FIB) technique using a Helios NanoLab 600i (FEI).

## 3. Results and discussion

### 3.1. Mo film deposited on (001)-oriented MgO substrate

#### 3.1.1. Formation of orientation variants

**Figure 1:**
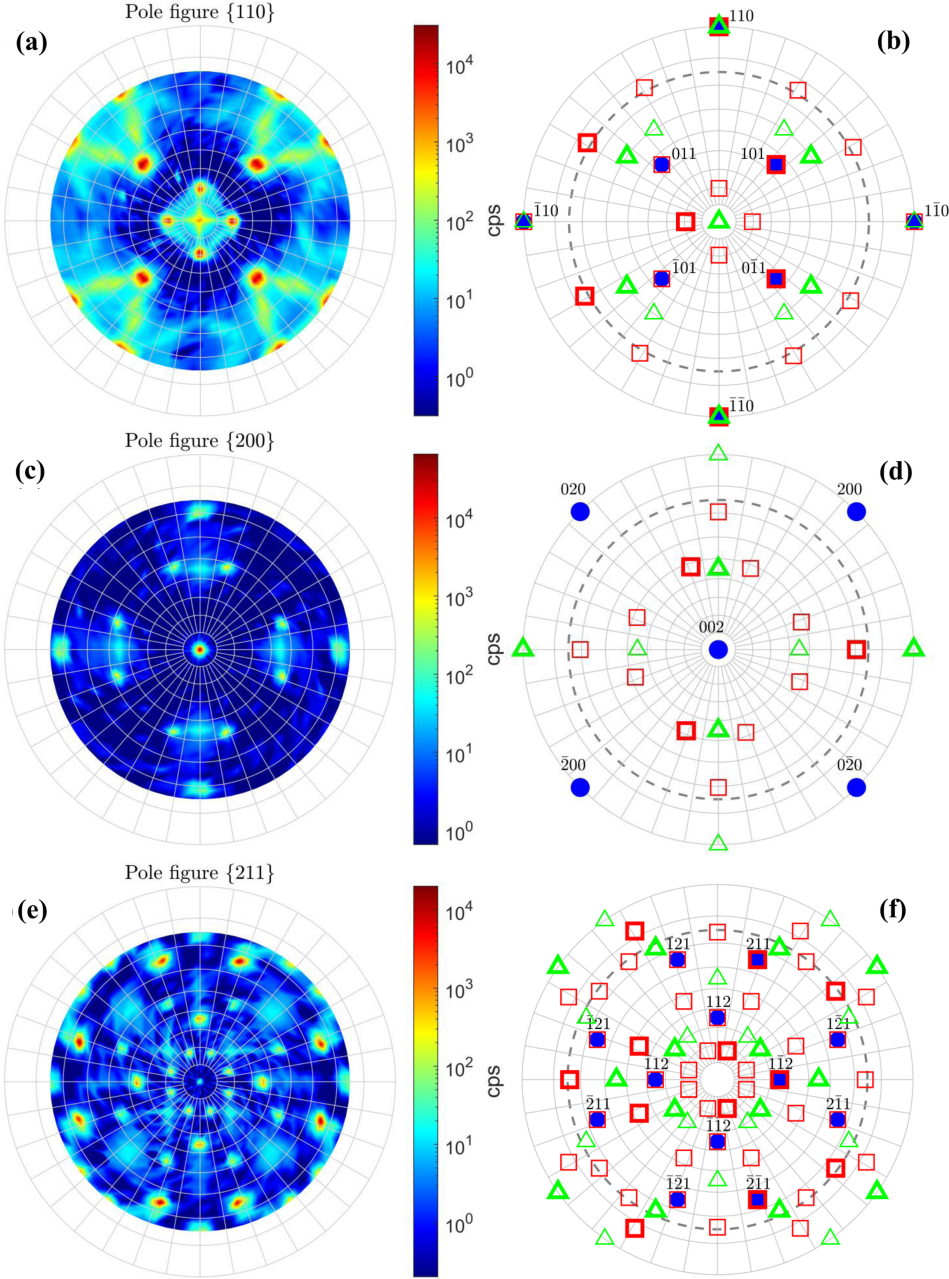
XRD {110}, {200} and {211} pole figures of the Mo film deposited on the (001)-oriented MgO substrate. Left panels show measured pole figures. Diffracted intensities are displayed in logarithmic scale. Right panels show positions of the individual poles calculated for the orientation relationships 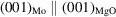 & 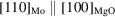 (matrix, filled blue circles), 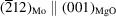 & 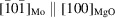 (twins, open red boxes) and 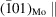

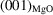
 & 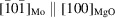 (minor variant, open green triangles). The poles corresponding to the primary orientation variants from equations (2*b*) and (2*c*) are highlighted by open symbols with bold edges. The orientation variants resulting from the fourfold rotation of the respective primary variant are plotted using open symbols with thin edges. The grey dashed circles in the right panels mark the maximum sample inclination (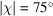) in the measured pole figures (left panels).

Analysis of the pole figures of the Mo film deposited on the (001)-oriented MgO substrate (Fig. 1 ▸) revealed that this film consists of three families of crystallites having the following primary orientation relationships to the substrate: 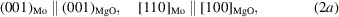

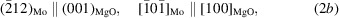

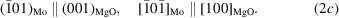
The orientation variant (2*a*[Disp-formula fd2]), which is called the *matrix* in further text, was expected to be the most probable one, as it preserves the crystallographic symmetry operations provided by the substrate (Rafaja *et al.*, 2002 ▸) and as it produces moderate lattice misfit between the substrate and the Mo film of about 5.4% (see Section 3.1.2). This expectation was confirmed by the high intensities of the matrix poles in the pole figures (Fig. 1 ▸).

The crystallite families obeying the orientation relationship (2*b*[Disp-formula fd2b]) are *twins* sharing the lattice planes {112} with the matrix. The smallest angle between the relevant twinning planes 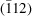, 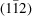, (112) and 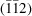 and the horizontal lattice planes (001) in the matrix is 35.3°, which is a half of the angle between the lattice planes 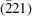, 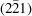, (221), 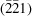 and (001). Thus, the twinning on the lattice planes {112} produces the orientation relationship 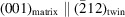, which follows from equations (2*a*[Disp-formula fd2]) and (2*b*[Disp-formula fd2b]). The combination of this ‘primary’ twinning process with a ‘reverse’ one that occurs on the twinning planes {112} that are parallel with the original ones results in the Mo grains switching their orientation along the sample surface normal direction between {001} and {221}.

**Figure 2:**
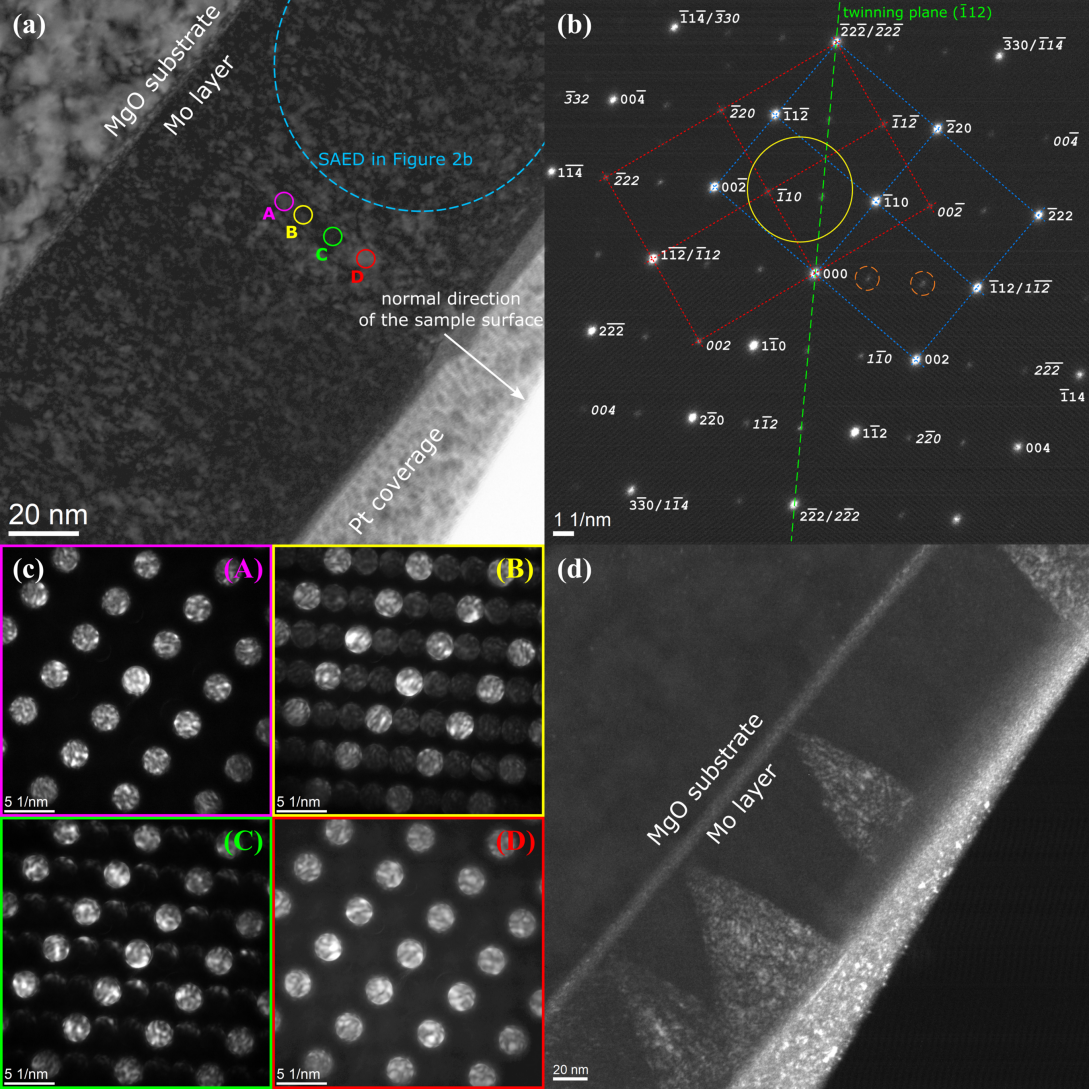
(*a*) Bright-field TEM image of the Mo film deposited on the (001)-oriented MgO substrate. The growth direction 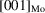 is marked by the white arrow. The Pt cover layer stems from the FIB sample preparation. (*b*) SAED pattern obtained from the area marked by the large dashed circle in panel (*a*). Diffraction spots from the matrix are labelled in normal script and diffraction spots from the twins in italics. Non-indexed diffraction spots (two of them are highlighted by small dashed orange circles) correspond to double reflections. (*c*) CBED patterns measured at the positions marked as A, B, C and D in panel (*a*). The zone axes of the SAED and CBED patterns are [110] for both matrix and twins. (*d*) Dark-field image taken using the diffraction spots enclosed by the solid yellow circle in panel (*b*), *i.e.*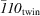 and the double reflection from the matrix/twin interface that is located between the diffraction spots 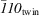 and 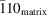.

**Figure 3:**
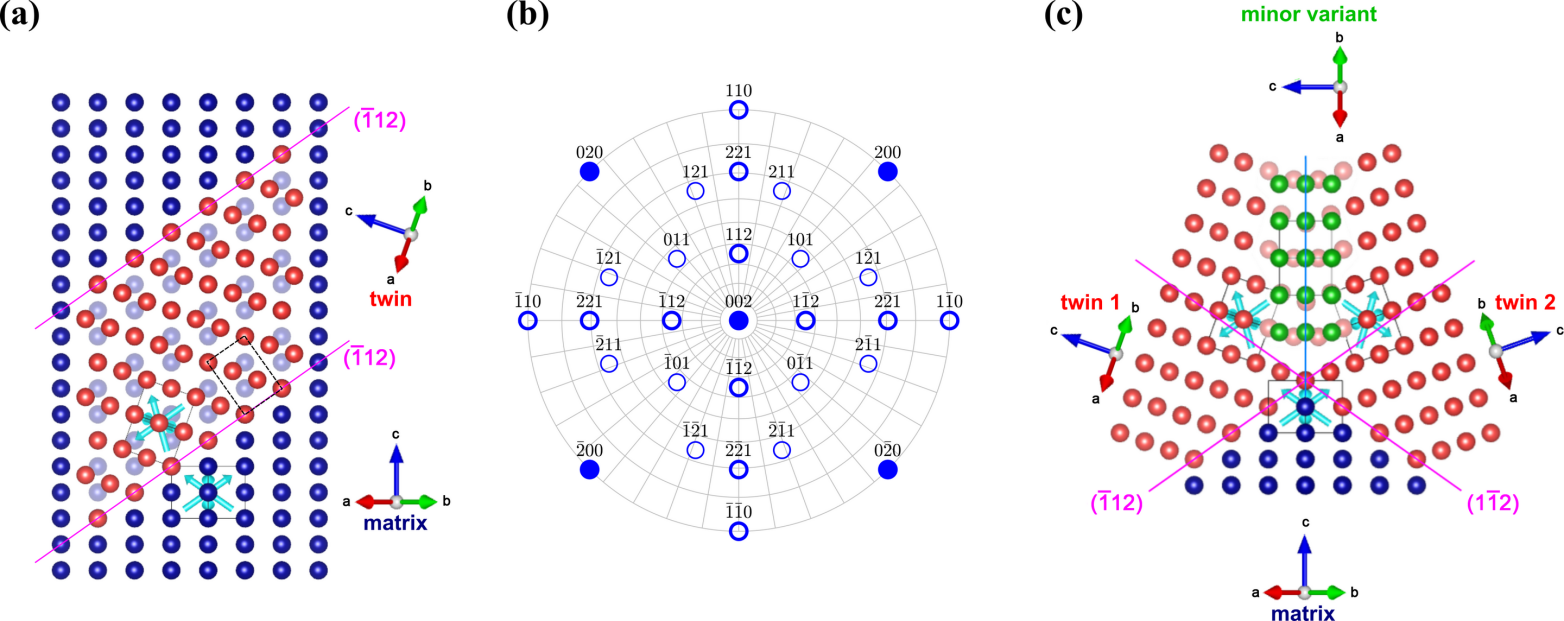
(*a*) Atomistic model of a single twin (red atoms) embedded in the Mo matrix (blue atoms). Semitransparent blue spheres show the original positions of Mo atoms in the non-twinned matrix. (*b*) Stereographic projection {001} (matrix orientation) illustrating orientations of the twinning planes {112} and positions of the poles {110}, {200} and {221} from the upper hemisphere. (*c*) Atomistic model of the interface between two twin variants growing on differently oriented twinning planes, *i.e.*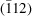 and 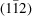 [see panel (*b*)]. Green atoms belong to the minor variant having the orientation from equation (2*c*). The atomistic models were drawn using *VESTA* (Momma & Izumi, 2011 ▸). In the structure models, the solid boxes mark the elementary cell of Mo. The Burgers vectors 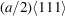 are depicted as cyan arrows. The dashed black box in panel (*a*) highlights the elementary cell of a superstructure oriented along the reciprocal space direction {211} (see text).

The presence of twins on the Mo lattice planes {112} was confirmed by SAED and CBED performed on a (110)-oriented cross section of the sample (Fig. 2 ▸). The SAED pattern [Fig. 2 ▸(*b*)] that was taken with a 90 nm slit from the area marked by the dashed circle in Fig. 2 ▸(*a*) shows intense matrix reflections, weak reflections stemming from a single twin variant and double reflections produced by the matrix/twin interfaces (Cayron, 2021 ▸). The double diffraction spots resemble reflections from a superstructure oriented along the {112} direction that has a periodicity of 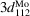, where 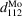 is the interplanar spacing of the Mo lattice planes {112}. An elementary cell of this superstructure is highlighted exemplarily by the dashed line in Fig. 3 ▸(*a*). The twinning plane 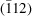 depicted in Fig. 2 ▸(*b*) is inclined by 35.3° from the interface between the MgO substrate and the Mo film, which is parallel to the (001) planes of Mo [*cf.* Fig. 3 ▸(*b*)].

The CBED patterns [Fig. 2 ▸(*c*)], which were acquired with a diameter of the incident electron beam of about 1–2 nm, confirm that the contrasts in Fig. 2 ▸(*a*) stem from the local presence (sub-panels B and C) and absence (sub-panels A and D) of the twins. This result indicates a high density and a small size of the twins. The dark-field TEM image [Fig. 2 ▸(*d*)], which was acquired simultaneously using the 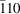 reflection from a single twin variant and a reflection stemming from the matrix/twin interface, shows that the single twin variant extends over several tens of nanometres. Thus, the twinning on the specific {112} plane occurs in a large part of the sample. The CBED patterns and local fast Fourier transformations (FFT) of the HRTEM image taken at the MgO/Mo interface reveal that the twins start to form in the vicinity of the substrate. However, a successive increase of the lateral extent of the regions containing twins, which is visible in the dark-field TEM image [Fig. 2 ▸(*d*)] and which was confirmed by CBED, indicates that the twinning probability increases with increasing distance from the substrate.

The atomistic models in Fig. 3 ▸ are based on results of the XRD pole figure measurements (Fig. 1 ▸), SAED and CBED (Fig. 2 ▸). In stereographic projection, the twinning illustrated in Fig. 3 ▸(*a*) follows the path 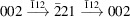 [Fig. 3 ▸(*b*)], which corresponds to the rotation of the matrix by 70.5° around the [110] axis. In the pole figure [Fig. 1 ▸(*b*)], such lattice rotation leads to the overlap of several poles {110} that belong to the matrix and twins. Additional twin variants form on crystallographically equivalent twinning planes (112), 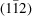 and 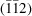 [*cf.* Fig. 3 ▸(*b*)]. This effect generates an apparent fourfold symmetry of the twins in the pole figures (Fig. 1 ▸) that complies with the fourfold symmetry of the matrix. The atomistic model from Fig. 3 ▸(*a*) suggests that the twins are fully coherent with the matrix in the twinning direction, if their length along a 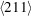 direction perpendicular to the respective twinning plane is equal to an integer multiple of 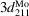. In other directions, the transition between twin and matrix is incoherent, as it must be mediated by dislocations. Most suitable are dislocations whose Burgers vector is parallel with the respective twinning plane, *e.g.* dislocations with the Burgers vector 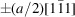 in the case of the twinning plane 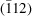.

The *minor* orientation variant that is described by equation (2*c*)[Disp-formula fd2c] corresponds to the rotation of the matrix by 90° around the 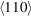 direction [Figs. 3 ▸(*b*) and 3 ▸(*c*)]. Thus, it also preserves the positions of one set of the {110} poles in the pole figure from Fig. 1 ▸(*b*), either (110) and 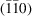 or 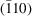 and 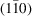. The orientation relationship between matrix and minor variant from Fig. 3 ▸(*c*) ensures parallelism of the lattice planes 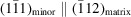 and 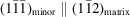, which makes the lattice planes 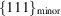 apparent twinning planes for the minor variant. This twinning follows the path 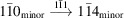 or 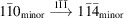. Parallelism of the lattice planes 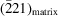, 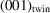 and 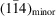 produces the 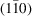 orientation of the minor variant through the twinning path 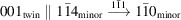. Moreover, the lat­tice planes {221} and {001} in body-centred cubic (b.c.c.) Mo have commensurate interplanar spacings, as 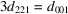. The same is also true for the lattice planes {114} and {110}, as 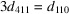. The parallelism of these lattice planes and their commensurate interplanar spacing facilitate the overlap of the reciprocal lattice points belonging to individual orientation variants and thus their coherence for diffraction experiments (Rafaja *et al.*, 2004 ▸).

In view of the above orientation relationships, the minor variant can appear at the interface between twin and matrix, where it terminates twins in the lateral directions. Alternatively, it can form at the interface between the twin variants which develop on crystallographically equivalent twinning planes {211}, in particular between twins that are mutually rotated by 180° around the normal direction to the sample surface [Fig. 3 ▸(*c*)]. In this case, the minor variant helps to ‘join’ the neighbouring twins. However, the adaptation of this minor variant to the surrounding twins requires small local lattice rotations (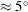), which are manifested as smeared intensity maxima belonging to the minor variant in the measured pole figures [Figs. 1 ▸(*a*), 1 ▸(*c*) and 1 ▸(*e*)]. In contrast to the first kind of minor variant, which can remain coherent with its neighbourhood, a misoriented minor variant disturbs the crystallographic coherence of the adjacent regions and makes the crystallites appear smaller from the point of view of the diffraction experiments (Rafaja *et al.*, 2004 ▸).

Comparison of the integral intensities of the non-overlapping poles in Fig. 1 ▸ revealed that the matrix dominates, having a volume fraction 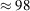
%, while the volume fractions of twins and minor variant are much smaller, being about 2% and 0.1%, respectively.

#### 3.1.2. Residual stress

The orientation relationship 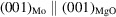 & 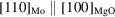 between the Mo matrix and the MgO substrates leads to a lattice misfit of
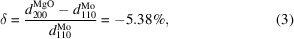
which was calculated following Wüstefeld *et al.* (2017 ▸) from the difference of the interplanar spacings of mutually parallel lattice planes 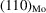 and 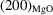 that are perpendicular to the Mo(001)/MgO(001) interface. The interplanar spacings were calculated using the lattice parameters 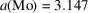
 Å (Jette & Foote, 1935 ▸) and 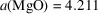
 Å (Hazen, 1976 ▸). As 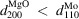 and 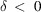, the Mo matrix is expected to be compressively stressed. However, it is very likely that the lattice strain induced by the lattice misfit is partly relieved, because the lattice misfit is rather high.

**Figure 4:**
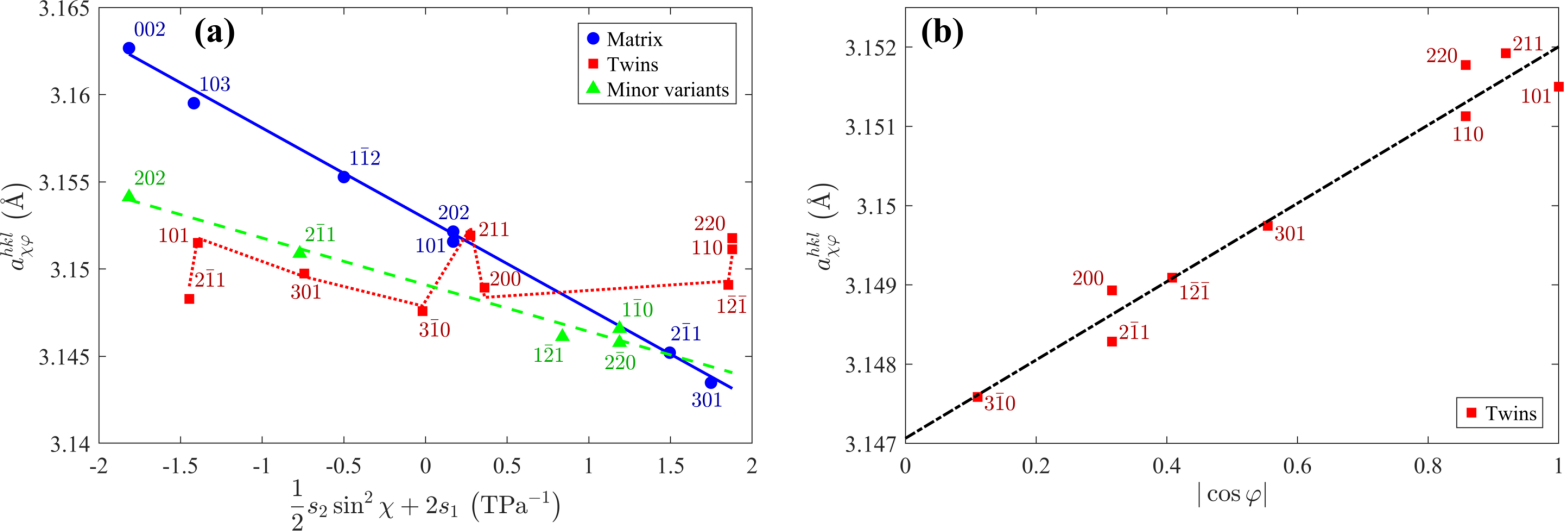
Lattice parameters of individual orientation variants of crystallites in the Mo film deposited on the (001)-oriented MgO substrate. Panel (*a*) shows the lattice parameters of all orientation variants as a function of 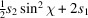 [*cf.* equation (7)]. Panel (*b*) illustrates the dependence of the lattice parameters of twins on 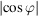 (see text for more details). Measured lattice parameters are plotted by symbols and the respective fits by lines.

In order to quantify the lattice strain relaxation, the remaining elastic lattice deformation in all orientation variants was concluded from residual stresses that were determined from the dependence of the measured lattice parameters 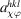 on the orientation of the diffraction vector in the sample coordinate system (Macherauch & Müller, 1961 ▸; Noyan & Cohen, 1987 ▸; Hauk, 1997 ▸; Welzel *et al.*, 2005 ▸): 
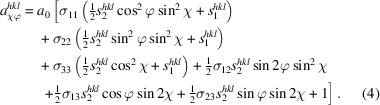
In equation (4), 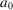 is the stress-free lattice parameter, 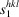 and 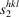 are the X-ray elastic constants (XECs), and *hkl* are the diffraction indices. According to Featherston & Neighbours (1963 ▸), the single-crystal elastic constants of Mo are 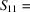 2.909 TPa^−1^, 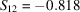
 TPa^−1^ and 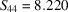
 TPa^−1^. As the corresponding Zener anisotropy ratio (Nye, 1985 ▸), 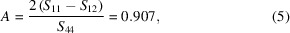
is not far from unity, Mo was regarded as an elastically isotropic material, and the XECs were calculated using Voigt’s model (Voigt, 1910 ▸): 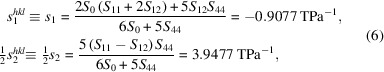
where 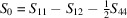. The lattice parameters 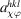 were calculated from the 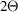 positions of the XRD lines that were measured using the crystallite group technique as described in Section 2. As can be seen in Fig. 4 ▸(*a*), the lattice parameters 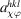 of the matrix and the minor orientation variant are independent of sample rotation (and thus of φ), and follow the *a* versus 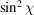 dependence (Noyan & Cohen, 1987 ▸; Perry *et al.*, 1992 ▸) 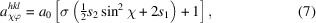
which corresponds to equation (4) with 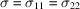 and 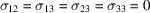. This finding indicates that the matrix and the minor orientation variant are under equiaxial in-plane stress. Refined parameters of the fit (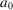 and σ) are given in Table 1 ▸. The remaining elastic deformation of the matrix calculated from the residual stress using 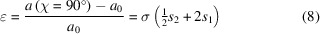
was −0.36%, which is approximately 15 times smaller than the lattice misfit of −5.38% from equation (3). This difference confirms a strong relaxation of the residual stress induced by the lattice misfit between the Mo matrix and the MgO substrate. In the minor variant, the residual stress is about two times smaller than that in the matrix (Table 1 ▸). Thus, also the lattice deformation of the minor variant is approximately two times smaller than that in the matrix.

In contrast to the matrix and minor variant, the lattice parameters of the twins depend strongly on 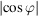 [Fig. 4 ▸(*b*)], while their 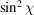 dependence is weak [Fig. 4 ▸(*a*)]. The absolute value in 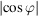 expresses equivalent behaviour of the twins formed on the planes {211} which are mutually rotated by 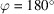. In Fig. 4 ▸(*a*), the 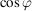-dependent term produces a characteristic scatter of the 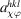 values. According to equation (4), the stress components 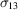 and/or 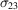 must be non-zero (Table 1 ▸), which means that the twins are under shear stress. In view of the different characters and amounts of residual stress in the individual orientation variants (mainly in matrix and twins), some mechanical interaction between them can be expected. This interaction produces complex local strain fields, which are responsible for contrasts within the CBED spots [Fig. 2 ▸(*c*)]. As the mechanical interaction between matrix and twins is not included explicitly in the stress model from equation (4), the refined parameters in Table 1 ▸ might be affected by systematic errors. This concerns in particular the stress-free lattice parameters, which differ slightly for individual orientation variants.

#### 3.1.3. Mechanism of residual stress relaxation

Residual stress analysis revealed a strong relaxation of the lattice strain induced by the lattice misfit between the film and substrate. The most effective relaxation mechanism of the lattice misfit is usually the formation of misfit dislocations having a Burgers vector that is located within the substrate/film interface. However, as the orientation of the interface is 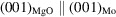 [see equation (2*a*[Disp-formula fd2])], the typical dislocations in b.c.c. crystal structures are less efficient, because their Burgers vectors 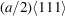 are inclined by 35.3° out of this interface [Fig. 3 ▸(*a*)]. Nevertheless, dislocations having these Burgers vectors can dissociate and produce stacking faults on the lattice planes {211} (Hirschhorn, 1963 ▸). This process facilitates twinning on the lattice planes {211}, which is a well known deformation mechanism in b.c.c. metals (Harding, 1967 ▸; Christian & Laughlin, 1987 ▸; Christian & Mahajan, 1995 ▸). Still, the high stacking-fault energy of Mo hinders the widening of the stacking faults (Hirschhorn, 1963 ▸; Natarajan & Van der Ven, 2020 ▸), which disqualifies *deformation* twinning as an efficient stress relaxation mechanism. Nonetheless, as two of the Burgers vectors 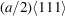 are located directly within the twinning plane {211}, their presence can contribute essentially to the reduction of the lattice strain, if the film contains *growth* twins. In the sample under study, this mechanism of the residual stress relaxation is corroborated by a low residual stress in the twins (Table 1 ▸) and by the prevailing shear stress component, the value of which is comparable to the yield strength of Mo, *i.e.* 385–410 MPa (Sturm *et al.*, 2007 ▸).

**Figure 5:**
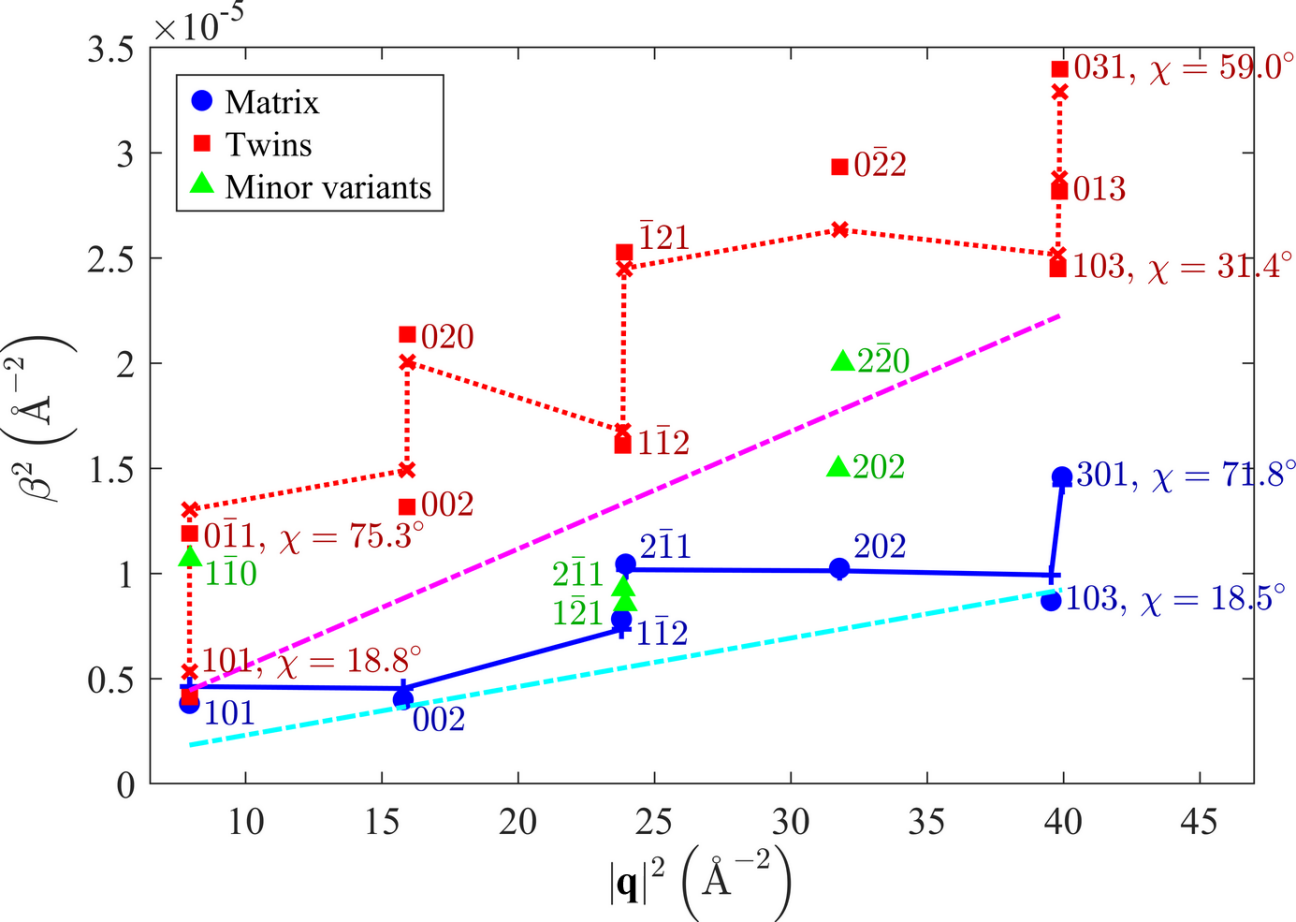
Dependence of the quadratic broadening of selected XRD lines (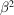) on the squared magnitude of the diffraction vector (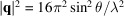) and on its inclination from the normal direction to the sample surface (χ) as measured for the Mo film deposited on the (001)-oriented MgO substrate. Solid symbols labelled by diffraction indices show measured integral breadths; scattered lines with crosses stand for the corresponding fit (see text). The straight lines represent the strain contribution to the XRD line broadening in the matrix (bottom) and in the twins (middle).

The presence of dislocations in individual orientation variants was examined through the analysis of the XRD line broadening [see *e.g.* Ungár *et al.* (1999 ▸)]. The measured integral breadths (solid symbols in Fig. 5 ▸) were obtained from the same data set as the line positions and lattice parameters in Section 3.1.2. A strong χ dependence of the line broadening indicates pronounced anisotropy of the size of coherently diffracting domains. For the sake of simplicity, the domain shape was approximated by a cylinder. The corresponding size contribution to the XRD line broadening followed the function 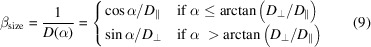
where 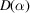 is the mean size of the cylinders along the diffraction vector, and α is the inclination of the diffraction vector from the cylinder axis. 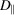 and 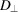 are the average length and diameter of the cylinders, respectively.

The strain contribution to the line broadening was assumed to be caused predominantly by dislocations and is described using (Wilkens, 1970 ▸; Ungár *et al.*, 1999 ▸) 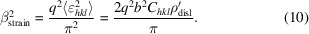
In equation (10), 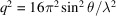 is the quadratic magnitude of the diffraction vector, 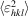 is the quadratic microstrain induced by dislocations, 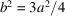 is the quadratic magnitude of the Burgers vector 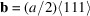 and 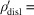

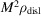
 is the apparent dislocation density containing the ‘true’ dislocation density 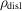 and the Wilkens factor *M*, which quantifies the shielding of the strain fields from neighbouring dislocations (Wilkens, 1970 ▸). 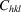 are the contrast factors of the dislocations (Klimanek & Kužel, 1988 ▸). According to Ungár *et al.* (1999 ▸), the dislocations’ contrast factors were approximated by their linear dependence on the cubic invariant, 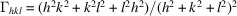: 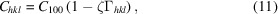
where 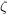 is the anisotropy of the dislocation contrast factors. For fitting, the strain broadening from equation (10) was replaced by 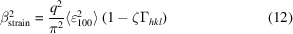
as described by Rafaja *et al.* (2013 ▸), where 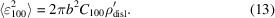
In analogy to equation (1), the total physical line broadening was calculated as 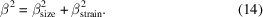
For the matrix, the best fit of the 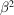 versus 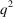 dependence (Fig. 5 ▸) was achieved with cylindrical domains that are oriented with their axis along the [001] direction, *i.e.* perpendicular to the sample surface. The length of the cylinders (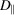) is equal to the thickness of the Mo film. Their diameter (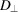) is smaller. The microstrain is practically isotropic (*hkl* independent), as ζ in equation (12) is equal to zero within experimental accuracy. For twins, the 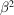 versus 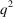 dependence in Fig. 5 ▸ was fitted best assuming cylindrical domains having the orientation 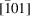. In the twin coordinate system, this direction is inclined by 19.5° from the normal direction 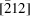 [*cf.* Fig. 1 ▸(*b*)]. The results of the fits are summarized in Table 2 ▸.

The dark-field TEM image [Fig. 2 ▸(*d*)] showing exemplarily the shape of partially coherent twinned regions reveals that these regions expand successively and asymmetrically in the lateral direction during the film growth. For XRD, this kind of the growth imitates an inclination of the ‘best coherence’ direction in the twins. As the mean lateral size of the coherently diffracting domains obtained from the XRD line broadening (Table 2 ▸) is smaller than the lateral size of the domains visible in the dark-field TEM image [Fig. 2 ▸(*d*)], it can be concluded that the film is more fragmented in the lateral direction than in the vertical direction. This means that the twinned regions are mutually coherent in an almost vertical direction as already discussed in Section 3.1.1 and illustrated in Fig. 3 ▸(*a*), while the mutual coherence of the neighbouring regions in the horizontal direction is interrupted by the transition between twin and matrix [Fig. 3 ▸(*a*)] or by the transition between two twin variants [Fig. 3 ▸(*c*)], because both transitions are assisted by dislocations.

The dislocation densities (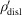) that were determined from microstrain (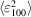) using equation (13) for matrix and twins are summarized in Table 2 ▸. The contrast factor, 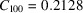, was calculated for screw dislocations with 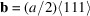 using the *ANIZC* routine (Borbély *et al.*, 2003 ▸). As already noted above, 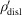 is not the true dislocation density, because it is not corrected for mutual shielding of the strain fields from neighbouring dislocations. Nevertheless, the estimated density of dislocations in the matrix is comparable to dislocation densities reported for pure Mo that was deformed at a shear strain of ∼5% (Loesch & Brotzen, 1967 ▸). The dislocation density is higher in twins than in the matrix, because the twins accommodate dislocations at their incoherent interfaces to the matrix (Fig. 3 ▸). These dislocations contribute to an almost complete relaxation of the residual stress in the twins (Table 1 ▸). For the minor variant, the values of the integral breadth are between the β values measured for matrix and twins (Fig. 5 ▸). However, the number of diffraction lines stemming from this orientation variant was not sufficiently high to be able to perform a detailed analysis like for matrix and twins.

On the basis of these results, it can be inferred that the relaxation of the residual stress in the Mo thin film deposited heteroepitaxially on the (001)-oriented MgO substrate is facilitated by twinning during the growth, by dislocations present in the twins that have their origin at the incoherent interfaces between twins and matrix, and by dislocations present in the matrix.

### 3.2. Mo film deposited on (011)-oriented MgO substrate

#### 3.2.1. Formation of orientation variants

**Figure 6:**
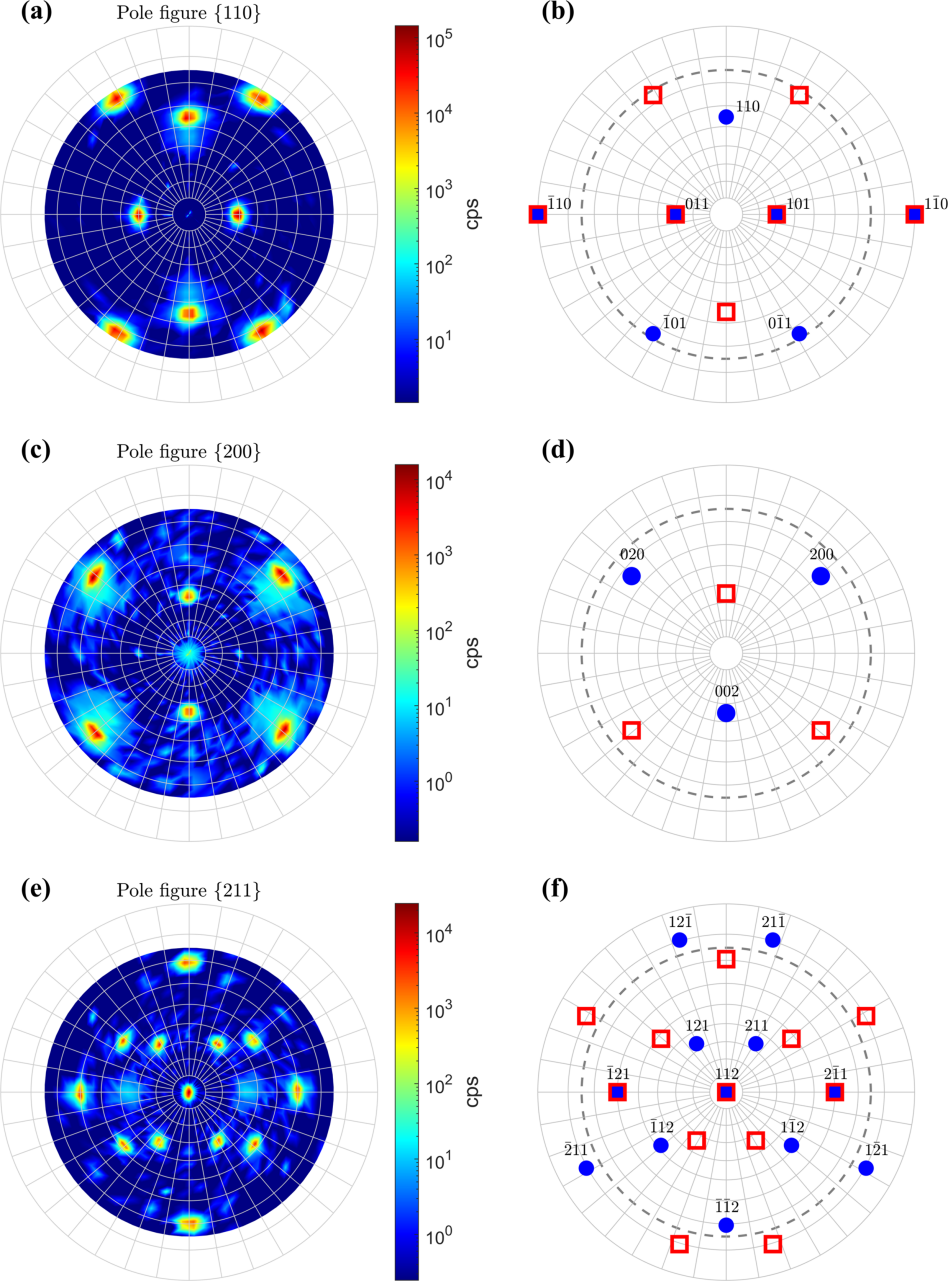
Pole figures of the Mo film deposited on the (011)-oriented MgO substrate. Left panels: measured pole figures {110}, {200} and {211} (from the top to the bottom). The diffracted intensities are shown in logarithmic scale. Right panels: corresponding pole figures simulated for the orientation relationships 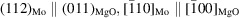 (blue filled circles) and 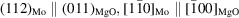 (red open boxes). The dashed circles in the right panel indicate the limit of the sample inclination (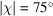) (see left panel). The weak and diffuse intensity maxima in the measured pole figures having no counterparts in the simulation stem from the tails of the MgO reflections.

**Figure 7:**
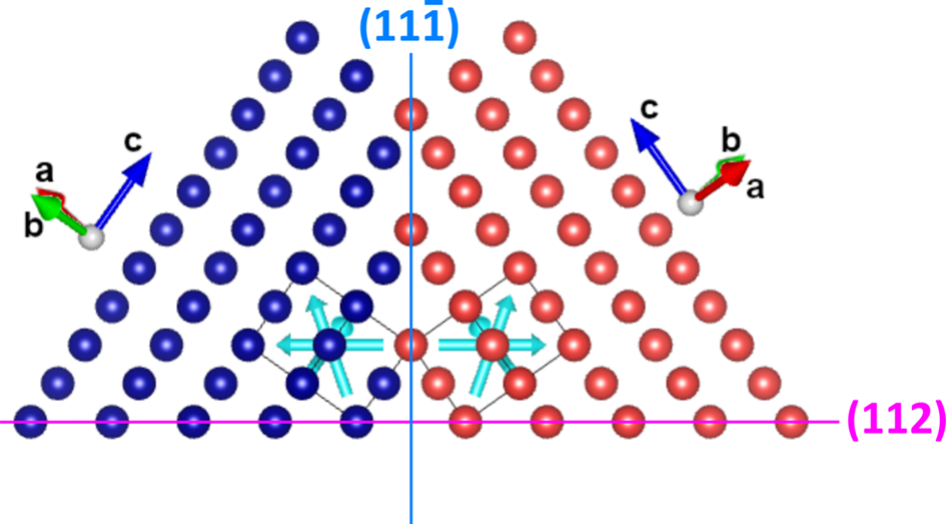
Atomistic model of the orientation variants present in the Mo film deposited on the (011)-oriented MgO substrate, depicted in the 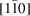 projection. The blue and red atoms correspond to the respective orientation variant. The cyan arrows mark the Burgers vectors 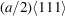. The model was drawn using *VESTA* (Momma & Izumi, 2011 ▸).

According to the pole figures shown in Fig. 6 ▸, the Mo film deposited on the (011)-oriented MgO substrate contains two orientation variants: 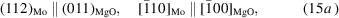

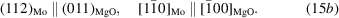
Evaluation of the integrated intensities of non-overlapping poles in the pole figures revealed that the volume fractions of the two orientation variants are similar, *i.e.* ∼57% for the orientation variant from equation (15*a*[Disp-formula fd15]) and ∼43% for the orientation variant from equation (15*b*). The existence of these two variants imitates the presence of a twofold axis and two mirror planes perpendicular to the sample surface like in the substrate. The atomistic model (Fig. 7 ▸) shows that the dislocations with the Burgers vectors 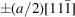 lie within the Mo(112)/MgO(011) interface in both orientation variants. Thus, they can act as misfit dislocations, reducing the lattice misfit between the substrate and the film. As the orientation variants are mutually rotated by 180° around their common [112] direction, the lattice plane 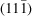, which is perpendicular to the Mo/MgO interface, is an idealized interface of differently oriented grains. However, as the {111} planes are not regular twinning planes in b.c.c. metals, these orientation variants are not expected to be crystallographically coherent.

**Figure 8:**
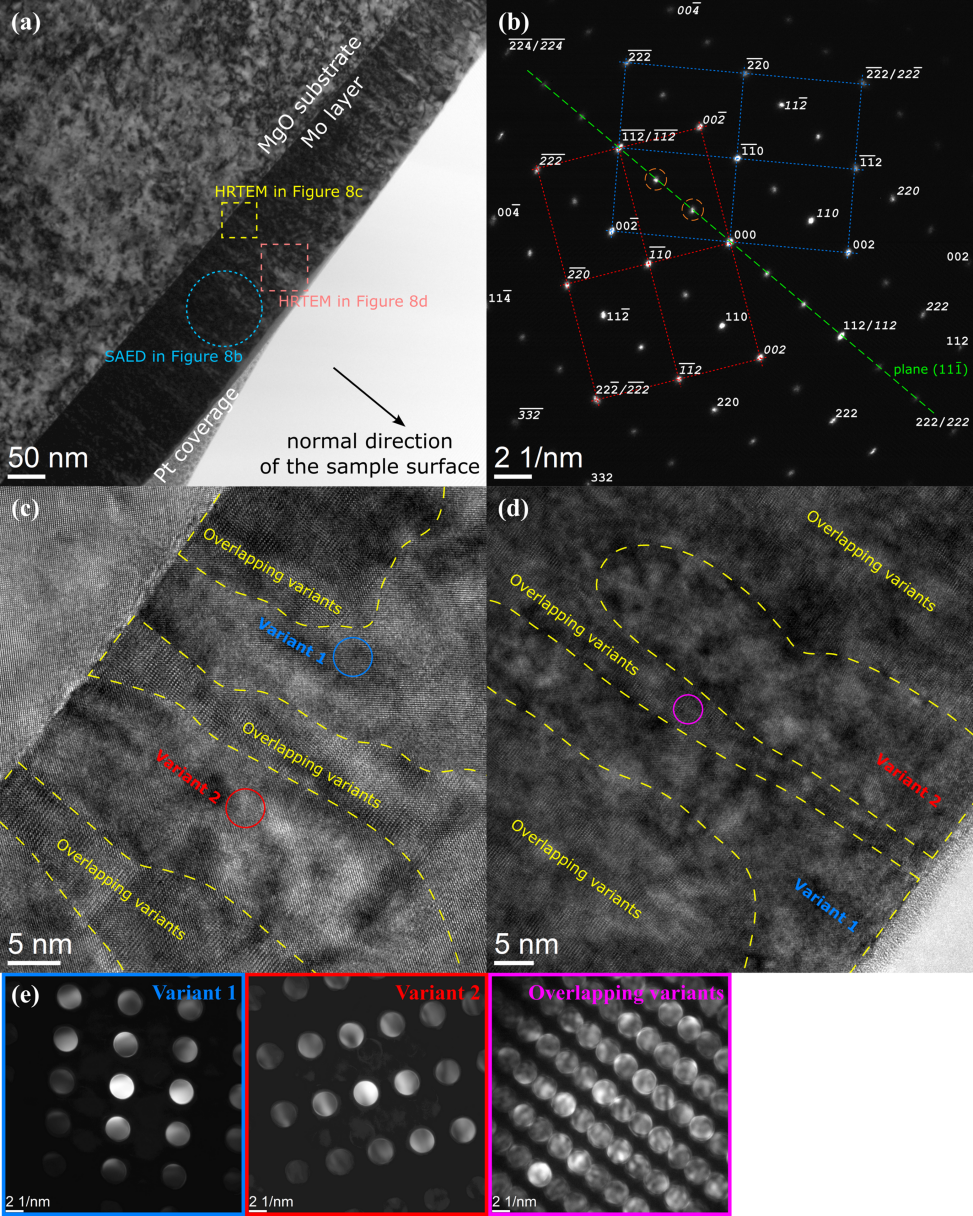
(*a*) Bright-field TEM image of the Mo film deposited on the (011)-oriented MgO substrate. The Pt cover layer stems from the FIB sample preparation. (*b*) SAED pattern obtained from the area marked by the large dashed circle in panel (*a*). Diffraction spots from the first orientation variant are labelled in normal script and diffraction spots from the second variant in italics. Non-indexed diffraction spots (two of them are highlighted by small dashed orange circles) correspond to double reflections. (*c*) and (*d*) HRTEM images of the areas marked by squares in (*a*). Circles mark the beam positions during the acquisition of the CBED patterns shown in panel (*e*). The zone axes of the SAED and CBED patterns are 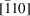.

The presence of the orientation variants from equations (15) and their mirroring by the lattice planes 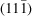 were confirmed by SAED/TEM (Fig. 8 ▸). Also in the SAED pattern of this sample [Fig. 8 ▸(*b*)], weak double diffraction spots (marked by dashed circles) can be seen, like in Fig. 2 ▸(*b*). The spacial distribution of the orientation variants was concluded from the local FFT of the HRTEM images that are shown in Figs. 8 ▸(*c*) and 8 ▸(*d*). Grains of both orientation variants start to grow directly at the substrate and are elongated in the vertical direction [112]. The lateral width of the grains is between 15 and 25 nm, which is less than the thickness of the TEM lamella (40–50 nm). Thus, different orientation variants can occur behind each other along the path of the electron beam, which explains the presence of large regions with overlapping variants in Figs. 8 ▸(*c*) and 8 ▸(*d*). Even the ‘single-variant’ regions contain bits of the respective second variant, as can be seen from the presence of additional weak CBED spots in Fig. 8 ▸(*e*). This overlap of the orientation variants produces double diffraction spots in Fig. 8 ▸(*b*), because it resembles stacking faults or twinning on the lattice planes (112) (*cf.* Fig. 7 ▸). Another explanation of the double diffraction spots in the SAED pattern is the occurrence of stacking faults on the lattice planes (112), which are produced by dislocations with 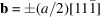 at the interface between different orientation variants.

#### 3.2.2. Residual stress

**Figure 9:**
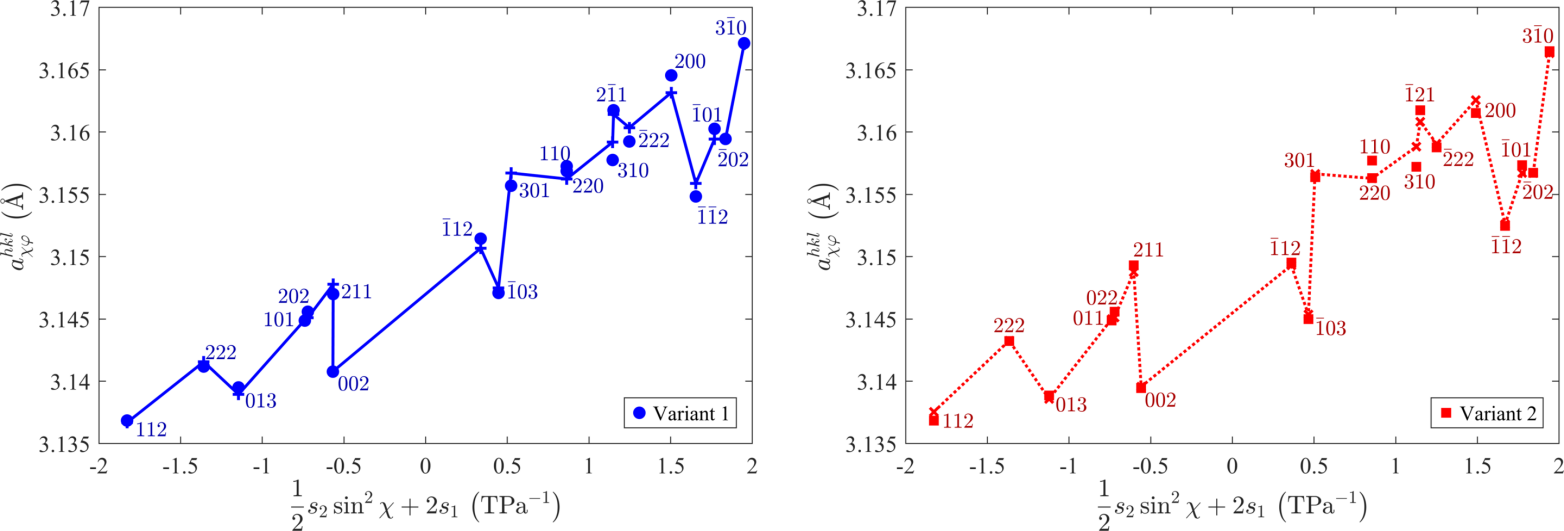
Lattice parameters of the orientation variants from equations (15*a*), left panel, and (15*b*), right panel, in the Mo film deposited on the (011)-oriented MgO substrate. Measured lattice parameters are plotted by solid symbols. Lines with crosses represent the least-squares fit using equation (4), in which the stress components 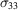 and 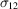 were kept zero.

The orientation relationships from equations (15) imply different lattice misfit at the Mo/MgO interface in the lateral directions 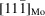 and 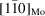: 
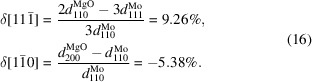
Consequently, the lattice strain in Mo is expected to be tensile in the 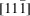 direction and compressive in the 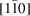 direction. Analysis of the residual stress using equation (4) confirmed that the residual stress is not equiaxial, as 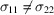 (Table 3 ▸). However, both components are positive. Moreover, both orientation variants are under significant shear stress, as can be seen from the non-zero 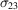 components. In Fig. 9 ▸, the shear stress is responsible for the scatter of the lattice parameters that is caused by their φ dependence, which is described by the term 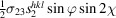 in equation (4) in this particular case. The φ dependence of 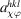, which was already discussed for the twins in the Mo film deposited on (001)-oriented MgO (Fig. 4 ▸), can be illustrated, for instance, on the lattice parameters 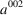 and 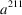 in Fig. 9 ▸(*a*). These lattice parameters have almost the same χ angle but different φ angles, which are equal to −90° and 58.5°, respectively [*cf.* Figs. 6 ▸(*d*) and 6 ▸(*f*)]. In can be concluded that shear stress occurs in such orientation variants, in which all crystallographic axes are inclined from the normal direction and from the substrate/film interface. This applies for twins in the Mo film deposited on (001)-oriented MgO and for both orientation variants in the Mo film deposited on (011)-oriented MgO.

In both orientation variants, the 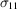 component of the stress tensor is oriented parallel to the crystallographic direction 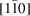, which is the projection direction in Fig. 7 ▸. The 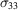 component is parallel to the vertical direction [112]. According to equations (15), these directions correspond to the first orientation variant (left grain in Fig. 7 ▸). Consequently, the φ angles are shifted by 180° for the second orientation variant. Thus, the functions 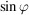 and 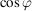 in equation (4) change their sign, which leads to negative values of the stress components 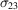 and 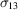 (Table 3 ▸). In contrast to the first sample (Table 1 ▸), the stress-free lattice parameters of the individual orientation variants in the second sample (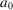 in Table 3 ▸) are almost identical within the experimental accuracy. This is because the orientation variants are mutually equivalent with respect to the lattice strain produced by the lattice misfit to the substrate and because the two variants are present in similar amounts (Section 3.2.1). Hence, the stress-free lattice parameters of the two orientation variants are affected equally by the mechanical interaction of differently oriented domains. Furthermore, the rather plain contrasts within the CBED spots from ‘separated’ orientation variants [Fig. 8 ▸(*e*)] suggest that a possible mechanical interaction between the orientation variants does not produce complex local strain fields.

#### 3.2.3. Mechanism of the residual stress relaxation

The measured pole figures from Fig. 6 ▸ confirm that the heteroepitaxy between Mo and (011)-oriented MgO produces an apparent twofold rotation axis [112] and mirror symmetry of the orientation variants in the Mo film. One of the mirror planes, 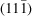, is displayed in Fig. 7 ▸. The other is 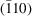. These symmetry operations imply a possible inequality of the stress components 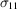 and 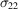, which was confirmed experimentally (Table 3 ▸). However, both measured stress components were positive, although the lattice misfits calculated for the 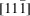 and 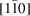 directions have different sign, 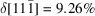 and 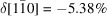 (Section 3.2.2). The remaining elastic lattice deformations calculated using equation (8) from the residual stress components 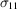 and 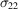 given in Table 3 ▸ are between 0.4% and 0.6%, which means that the lattice strain caused by the lattice misfit is strongly relaxed. The presence of a significant shear stress component 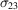 suggests that the slip on one of the b.c.c. lattice planes containing the Burgers vectors 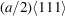, for instance {110}, {112} or {123} (Lee *et al.*, 1999 ▸; Krenn *et al.*, 2001 ▸), is an important mechanism of stress relaxation. Several of these lattice planes are inclined by 30–60° from the Mo/MgO interface, where the primary lattice strain is induced by the lattice misfit, which facilitates the slip in terms of Schmid’s law. The inclinations of the lattice planes {110} and {112} are visible in Figs. 6 ▸(*b*) and 6 ▸(*f*).

**Figure 10:**
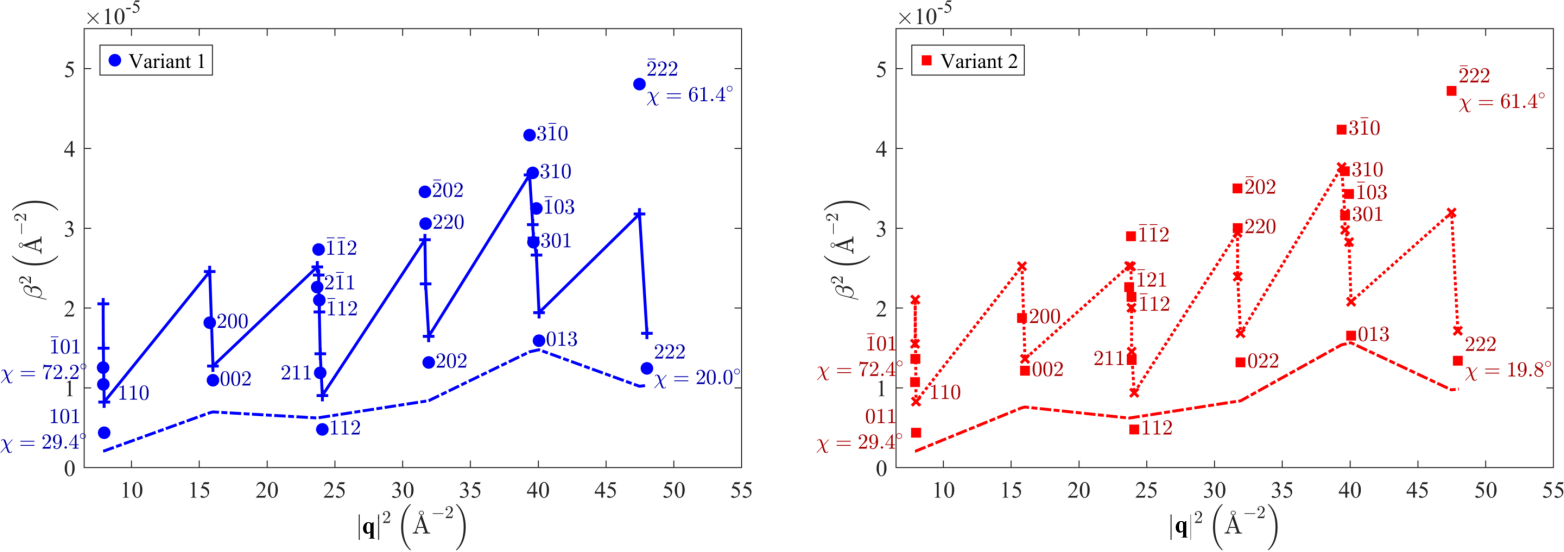
Dependence of the quadratic broadening of selected diffraction lines (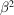) on the squared magnitude of the diffraction vector (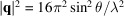) and on the inclination of the diffraction vector from the normal direction to the sample surface (χ) as measured for the Mo film deposited on the (011)-oriented MgO substrate. Symbols labelled by diffraction indices represent measured integral breadths. Solid and dashed lines show the corresponding fit using equations (9), (12) and (14). The dash–dotted lines at the bottom of the plots illustrate the strain contribution [equation (12)] to the XRD line broadening.

Fitting of the XRD line broadening (Fig. 10 ▸) using equations (9)–(14) confirmed a strong anisotropy of the crystallite size, which was already expected from the HRTEM images [Figs. 8 ▸(*c*) and 8 ▸(*d*)], and verified the presence of the dislocation-induced microstrain (Table 4 ▸). Like in the previous sample, the coherently diffracting domains were assumed to possess a cylindrical shape. In the vertical direction, they are extended throughout the whole film. Their size in the lateral direction is about 20 nm, because the crystallites belonging to different orientation variants are usually mutually incoherent. They only become coherent when their reciprocal lattice points overlap (Rafaja *et al.*, 2004 ▸). This is true for matching poles or reflections 110, 111 and 211 in Figs. 6 ▸(*b*), 6 ▸(*f*) and 8 ▸(*b*). This additional coherence intensifies the χ dependence of the XRD line broadening in Fig. 10 ▸, because it makes the crystallites apparently larger in some non-vertical directions, while their size in the lateral direction is still limited by the distance between the ‘incoherent’ 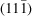 planes (Fig. 7 ▸). The different degree of the crystallographic coherence of individual orientation variants in different reciprocal space directions is the main reason for the partial disagreement between the measured and calculated line broadening in Fig. 10 ▸, which is largest for the diffraction lines 222. Still, despite these coherence issues, the sizes of the coherently diffracting domains determined from the XRD line broadening (Table 4 ▸) are in good agreement with the domain sizes estimated from the HRTEM images [Figs. 8 ▸(*c*) and 8 ▸(*d*)].

In contrast to the Mo film deposited on the (001)-oriented MgO substrate, the anisotropy of the microstrain, which is quantified by the parameter ζ in equation (12), must be considered for the Mo film deposited on (011)-oriented MgO. This difference can be explained by the different character of the dislocations in individual samples. According to Ungár *et al.* (1999 ▸), ζ is equal to 1.8 for screw and to −1.1 for edge dislocations, assuming the anisotropy ratio 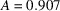 [equation (5)] and the stiffness ratio 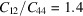 that were calculated using the elastic constants from Section 3.1.2 (Featherston & Neighbours, 1963 ▸). On the basis of the microstrain anisotropy determined from the measured XRD line broadening using equation (12), *i.e.*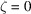 for the first sample and 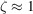 for the second sample (Table 4 ▸), it can be concluded that the dislocations in both samples have a mixed character. Nevertheless, the ratio between the edge and screw dislocation fraction is different in individual samples. The mixed dislocations present in the matrix and in the twins of the Mo film deposited on (001)-oriented MgO have predominantly edge character, whereas the dislocations in the Mo film deposited on (011)-oriented MgO have a dominant screw component. In both orientation variants of the second sample, the dislocation density (Table 4 ▸) is comparable to the dislocation density in the twins of the first sample (Table 2 ▸), while the density of dislocations in the matrix of the first sample is significantly lower.

### 3.3. Factors affecting the microstructure of Mo grown heteroepitaxially on MgO

**Figure 11:**
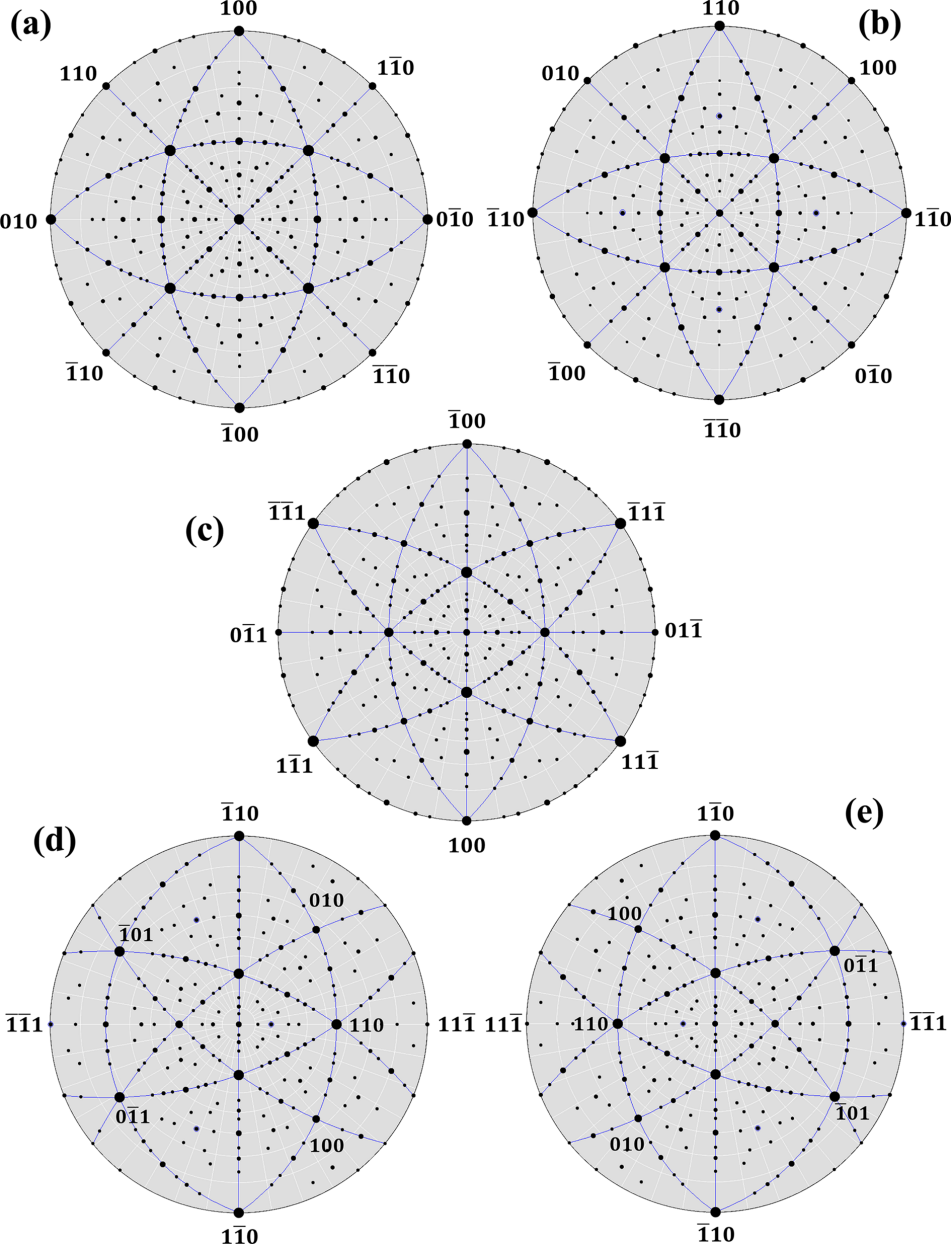
Stereographic projections (001) of face-centred cubic (f.c.c.) MgO (*a*) and (001) of b.c.c. Mo (*b*) having the orientation relationship 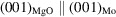 & 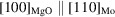. Lines mark zones {110} (*a*) and {111} & {100} (*b*). Stereographic projections (011) of f.c.c. MgO (*c*) and (112) of b.c.c. Mo (*d*, *e*) having the orientation relationships 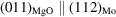 & 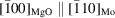 (*d*) and 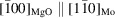 (*e*). Lines mark zones {110} & {100} (*c*) and {111} & {100} (*d*, *e*). The stereographic projections were plotted using *WinWulff* (Weber, 2018 ▸).

Heteroepitaxial growth of thin films on single-crystalline substrates is usually controlled by the crystallographic symmetry of the substrate and by the deformation energy accumulated in the film (Grünbaum, 1975 ▸; Geiesche *et al.*, 1988 ▸; Brune & Kern, 1997 ▸). For the Mo films under study, the interplay of these two factors can be illustrated using stereographic projections of the counterparts and their lattice misfit. The stereographic projections depicted in Figs. 11 ▸(*a*) and 11 ▸(*b*), which were simulated for the orientation relationship 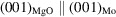 & 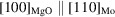 from equation (2*a*[Disp-formula fd2]), show matching positions of several poles at the rim of the pole figures. These poles correspond to the lattice planes with the zone axis [001], which are perpendicular to the Mo(001)/MgO(001) interface. Identical positions of these poles in both stereographic projections mean that the corresponding lattice planes in MgO and Mo are mutually parallel. Hence, not only are the lattice planes 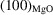 and 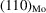 parallel to each other but also 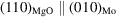, 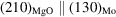, 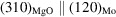, 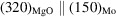 and others. These sets of parallel lattice planes produce the same lattice misfit of −5.38%.

Comparison of interplanar spacings of other, non-parallel lattice planes revealed that a smaller lattice misfit would be achieved for the lattice planes 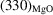 & 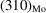 and 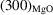 & 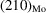, having a lattice misfit of about −0.3%, or for the lattice planes 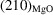 & 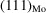 and 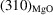 & 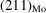, which would produce a lattice misfit of about −3.7%. From this finding, it can be concluded that the orientation relationship 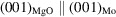 & 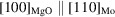 is established primarily to preserve the symmetry operations of the substrate, which are the fourfold axis (001) perpendicular to the Mo/MgO interface and the mirror planes {100} and {110} [Figs. 11 ▸(*a*) and 11 ▸(*b*)]. Following this orientation relationship to the substrate, the Mo film must accept a large compressive stress, which is, however, released through dislocations and growth twins. The twinning, which occurs typically on the lattice planes {211} in b.c.c. structures, is enabled by a large number of these lattice planes and by their favourable inclinations with respect to the Mo/MgO interface [Fig. 3 ▸(*b*)], which facilitate the slip on the lattice planes {211}. As two of the Burgers vectors 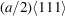 lie in the {211} plane, the corresponding dislocations can easily compensate the atomic disorder between the Mo matrix and the twinned regions [Figs. 3 ▸(*a*) and 3 ▸(*c*)].

For the orientation relationship 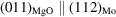 & 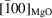

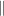

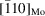
 or 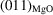

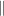

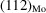
 & 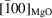

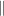

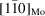
 from equations (15), the symmetry operations of the substrate, which are the twofold axis [011] and the mirror planes 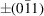 and 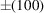 [*cf.* Fig. 11 ▸(*c*)], are preserved not by individual orientation variants but only by their combination, which reproduces well the symmetry operations of the substrate [Figs. 11 ▸(*d*) and 11 ▸(*e*)]. Still, the poles in the stereographic projections of MgO and Mo coincide much less than for the orientation relationship 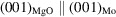 & 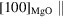

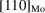
. The mutually parallel lattice planes with the zone axes 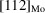 and 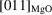 are 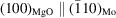 and 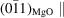

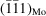
 only. The lattice misfit in the perpendicular in-plane directions 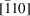 and 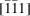 is −5.38% and 9.26%, respectively. However, this lattice misfit is reduced by the misfit dislocations with 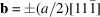, which lie within the Mo(112)/MgO(011) interface.

Although the lattice strain induced by the lattice misfit was largely relaxed, relatively high residual stress was found in both samples. The largest values of the residual stress tensor were always associated with the normal stress components 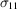 and 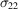. The presence of shear stresses that are approximately equal to the yield strength of Mo, 385–410 MPa (Sturm *et al.*, 2007 ▸), argue for slip as a possible relaxation mechanism. Because of the large lattice misfit between the Mo film and MgO substrate in both samples, the films are assumed to grow in the form of islands in the early stages of the deposition process (Lozovoy *et al.*, 2020 ▸). Later on, these islands coalesce, which may produce a tensile in-plane component of the residual stress in the film (Nix & Clemens, 1999 ▸). Small lateral crystallite size, which is an expectable consequence of the island growth, was confirmed by XRD and transmission electron microscopy.

## 4. Conclusions

Thin molybdenum films were deposited heteroepitaxially on single-crystalline MgO wafers with the dominant orientation relationships 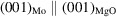 & 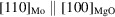 and 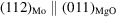 & 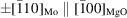. It was shown that the orientation of the film is controlled mainly by the conformity of the symmetry operations of the counterparts. Combination of X-ray diffraction utilizing the crystallite group technique with electron diffraction and transmission electron microscopy revealed that the residual stress in the films, which is induced by the lattice misfit between the film and the substrate, is largely relieved through the formation of dislocations and twinning. The activation of the respective strain relaxation process depends on the film orientation and on the orientation of the film/substrate interface. In the case of the Mo(001)/MgO(001) interface, the growth twinning on the Mo lattice planes {211} was the dominant mechanism of lattice strain relaxation in the film. Dislocations occurred mainly at the boundaries between twins and matrix. For the orientation relationship 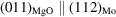, the lattice strain was relieved mainly via dislocations. For both orientation relationships under study, the deformation energy was additionally reduced through the small lateral size of the Mo grains.

## Figures and Tables

**Table 1:** Stress-free lattice parameters 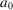 and relevant components of the residual stress tensor (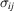) that were obtained from the least-squares fit of the measured lattice parameters from Fig. 4 by equation (7) or (4) For the matrix and minor variant, the stress components 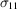 and 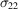 were coupled. Zero components of the residual stress were not refined.

Mo variant	*a*_0_ (Å)	σ_11_ = σ_22_ (GPa)	σ_13_ (GPa)	σ_23_ (GPa)
Matrix	3.1529 (2)	−1.65 (2)	0	0
Twins	3.1499 (4)	0	0.24 (5)	0.08 (7)
Minor	3.1491 (5)	−0.85 (4)	0	0

**Table 2:** Diameter (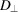) and length (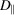) of coherently diffracting domains with cylindrical shape, dislocation-induced squared microstrain (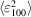) and apparent dislocation density (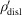) in the Mo film deposited on (001)-oriented MgO The direction of the cylinder axis, 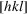, is related to the crystallographic orientation of the respective variant.

Mo variant	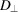	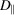	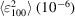	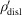
Matrix	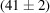 nm	100 nm 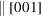	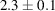	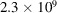 cm^−2^
Twins	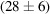 nm	100 nm 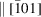	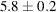	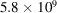 cm^−2^

**Table 3:** Stress-free lattice parameters 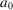 and non-zero components of the residual stress tensor (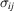) obtained from the least-squares fit of the measured lattice parameters from Figs. 9(*a*) and 9(*b*) by equation (4) The stress components 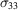 and 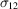 were not refined but kept zero.

Mo variant	*a*_0_ (Å)	σ_11_ (GPa)	σ_22_ (GPa)	σ_23_ (GPa)	σ_13_ (GPa)
Variant 1	3.1495 (1)	2.6 (1)	2.0 (1)	0.28 (7)	0.04 (6)
Variant 2	3.1492 (2)	2.6 (1)	1.66 (8)	−0.41 (6)	−0.02 (5)

**Table 4:** Diameter (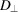) and length (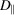) of coherently diffracting domains with cylindrical shape, dislocation-induced microstrain 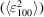, strain anisotropy 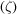 and apparent dislocation density 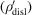 in the Mo film deposited on (011)-oriented MgO The direction of the cylinder axis is [112] (vertical).

Mo variant	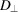	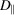	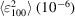	ζ	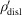
Variant 1	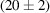 nm	100 nm	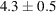	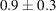	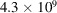 cm^−2^
Variant 2	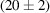 nm	100 nm	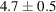	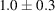	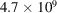 cm^−2^

## Data Availability

The data supporting the results are available upon request. A MATLAB routine for fitting 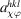 and 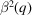 is available on GitHub (https://github.com/PetrCejpek/PSB_GUI).
